# Parkinson’s Disease in Romania: A Scoping Review Protocol

**DOI:** 10.3390/brainsci11020251

**Published:** 2021-02-17

**Authors:** Elena Cecilia Rosca, Raluca Tudor, Amalia Cornea, Mihaela Simu

**Affiliations:** 1 Department of Neurology, Victor Babes University of Medicine and Pharmacy Timisoara, Eftimie Murgu Sq. no. 2, 300041 Timișoara, Romania; tudor.raluca@yahoo.com (R.T.); amalia.cornea@yahoo.com (A.C.); mihaelasimu6713@gmail.com (M.S.); 2 Department of Neurology, Clinical Emergency County Hospital Timisoara, Bd. Iosif Bulbuca no. 10, 300736 Timisoara, Romania

**Keywords:** Parkinson’s disease, Romania, scoping review, protocol

## Abstract

Parkinson’s disease (PD) is a significant cause of disability, with a fast-growing prevalence. This review will summarize the epidemiological and clinical data in Romania and the interventions and diagnostic approaches used in this Eastern European country. This scoping review will primarily follow the recommendations on the scoping review methodology made by the Joanna Briggs Institute. In order to answer our research questions, we will search four databases using appropriate search terms. We will use pre-defined inclusion criteria and the data of eligible studies will be extracted in a standardized form. Results will be reported following the Preferred Reporting Items for Systematic reviews and Meta-Analyses extension for Scoping Reviews (PRISMA-ScR). The proposed scoping review will map the evidence on PD in Romania through a literature review, focusing on epidemiology, clinical characteristics, interventions, and diagnosis, contributing to PD research advancement. We will provide information for policy-makers, public health specialists, and clinicians.

## 1. Introduction

Parkinson’s disease (PD) is a significant source of disability. As the life expectancy increases, the number of PD individuals is expected to increase. Therefore, more effective treatment strategies are needed to address this burden. In addition, to examine PD trends, it is essential to gather data on the incidence and prevalence of the disease, especially in regions in which little data are available [1].

Romania is a middle-income Eastern European country with a population of approximately 19 million people, with 17.8% being over 65 years old. It is estimated that in Romania, there are over 72,000 patients with PD [2].

The latest research estimates that approximately 6 million people are diagnosed with PD worldwide. In Europe, there are an estimated 1.2 million PD patients, with the disease affecting 1% of the population over the age of 60 [1]. Although the research and care of patients with movement disorders, including PD, has made considerable progress in recent years, data from this Eastern European country have still not been fully uncovered.

A recent systematic analysis of epidemiological studies estimated the global, regional, and country-specific prevalence and years of life lived with disability for PD from 1990 to 2016. Authors produced estimates of the burden (prevalence, deaths, and disability-adjusted life-years (DALYs)). In Romania, they reported the following data: 1605 (95% uncertainty intervals UI 1201 to 2110) PD patients died and the prevalence of PD was estimated to be 40,517 (95% UI 31,427 to 50,995), with 23,144 (95% UI 17,467 to 30,057) disability-adjusted life-years (DALYs) [1]. The percentage change between 1990 and 2016 in age-standardized rates was 8.1% (95% UI −2.4 to 20.3) for PD deaths, with a prevalence percentage change in age-standardized rates of 10.2% (95% UI 4.5 to 15.5) and a DALYs change of 9.2% (95% UI 0.2 to 20.0) [1].

Observing the trends over time and understanding the epidemiology of PD is essential to identify the success or failures of intervention efforts. Therefore, there is a significant need to obtain all available data in Romania to map the severity of the problem accurately. Understanding the current situation will provide useful insight into the country’s context, assisting researchers, healthcare professionals, and policy-makers in decision-making. It will also help in developing future research strategies and designing and implementing programs to reduce the burden of PD in Romania.

The present scoping review aims to provide a comprehensive understanding of existing literature by reporting data on PD epidemiology, clinical characteristics, interventions, and diagnostic challenges in Romania by reviewing all published studies. Unlike classic systematic reviews that address relatively precise questions, the present scoping review will offer a much larger perspective on Romania’s PD research gaps. It aims to map and examine emerging evidence when it is still unclear what other, more specific research questions can be posed and valuably addressed. Therefore, a scoping review approach is a much needed and suitable method to assess the current situation of PD in Romania [3].

## 2. Materials and Methods

There are over 20 methods in the area of evidence synthesis [4,5]. We concluded that our specific synthesis method should be a scoping review based on our questions and objectives. We also used an online tool that helps authors to decide which knowledge synthesis method could be the most appropriate for their specific research question (https://whatreviewisrightforyou.knowledgetranslation.net/) [3,6]. As a pilot project, the current version of the tool only identifies methods for the knowledge synthesis of quantitative studies. By answering a series of five questions, authors are guided on the best type of knowledge synthesis to conduct in order to ensure that research goals are met. The tool is accompanied by an explanation and elaboration document which users can refer to for further assistance [7]. 

### 2.1. Research Questions

In order to identify significant problems to be addressed, we considered various aspects of PD in Romania. Therefore, we developed research questions defining clearly the Population, Concept, and Context (PCC) of the review [8]. 

Four research questions were identified to guide the present scoping review:

- What are the published data on the epidemiology of PD in Romania?
- What clinical aspects have been investigated in Romanian PD patients?
- What are the interventions introduced in Romania to reduce the burden of PD and improve the patient’s care and quality of life?
- Which are the diagnostic tests used in PD patients in Romania?

### 2.2. Search Strategy and Eligible Studies

We developed a broad search strategy based on the PCC mnemonic recommended for scoping reviews by the Joanna Briggs Institute [8]. 

We will perform a computerized bibliographic search on the following databases: MEDLINE/PubMed, EMBASE, Scopus, and Web of Science. We will also check reference lists of all relevant research papers to identify possible additional studies.

To ensure that we capture the maximum number of articles related to our research questions, we performed a scoping search. We found in the four databases a total number of 1133 citations. 

Our search strategy will use the following keywords: “Parkinson’s disease” [MeSH] AND “Romania.” These search terms are for PubMed. Searches in other data sources will use similar versions of these terms, appropriate for each database. We will not use search filters because we aim to generate a broad list of studies that would be suitable for answering our research question. Additionally, we will not apply any language restrictions. We may contact the authors of the selected primary studies or reviews for further information if this is relevant.

### 2.3. Study Selection

According to the PCC mnemonics, the present scoping review will include all human studies reporting research on adults (over 18 years old) with at least 10 participants (P), investigating the epidemiology, clinical characteristics, interventions, or diagnostic tests in PD patients (C) that were conducted in Romania (C). 

Therefore, we will include all studies recruiting participants that meet the UK Brain Bank criteria for PD [9], or the MDS clinical diagnostic criteria for PD [10], investigating motor and non-motor features of the disease. Additionally, studies investigating complications of PD treatment (e.g., peripheral neuropathy) will be included. 

We will not set limits on publication date, study design, or setting. However, we will include only quantitative studies; qualitative studies will be excluded because we do not aim to explore barriers and facilitators for an intervention. We will include both primary studies and systematic reviews if available. 

We will exclude studies with patients with secondary Parkinsonism (e.g., vascular, toxic, drug-induced, or post-infectious) or atypical Parkinsonism (e.g., corticobasal degeneration, Lewy body dementia, progressive supranuclear palsy, or multiple system atrophy).

The review will follow a two-stage screening process. First, two authors will review the title and abstract of all identified papers independently to assess the study’s eligibility based on the inclusion and exclusion criteria. Any paper that is considered eligible by either or both reviewers will be included for a full-text assessment. In the second stage, two authors will independently screen the full text of the selected articles. Disagreements will be solved by a third reviewer with expertise in the domain. 

### 2.4. Extraction of the Results

We will chart the results in order to provide readers with a logical descriptive summary of the results. A draft charting table was developed at the protocol stage to collect essential information about the source, such as the author, year of publication, where the study was conducted, aims of the study, study sample size, methods of the research, and results or findings relevant to our review questions. Depending on the study type, if applicable we will extract other additional data. 

For intervention studies, the information will be grouped into three categories: pharmacological, non-pharmacological, and a combination of pharmacological and non-pharmacological interventions. We will also chart data on the comparator and other details such as the duration of the intervention and outcomes. 

We will extract data on the test, comparator, and results for diagnostic test studies, including parameters such as sensibility, specificity, and other aspects (e.g., psychometric properties). 

The chart tables may be further refined and updated if it becomes apparent that additional unforeseen data can be usefully charted. Nonetheless, we will produce initial piloted data extraction forms on three studies from each category of the review questions to ensure all relevant results are extracted. 

Two independent reviewers will extract data into the final forms. A third reviewer will solve any discrepancies. 

### 2.5. Reporting the Results and Summarizing the Findings

The results of the search will be presented following the PRISMA-ScR recommendations [11,12]. 

We will provide a descriptive numerical summary, presenting the characteristics of the included studies. The results will be classified under the main conceptual categories, such as “epidemiology”, “clinical characteristics”, “interventions”, and “diagnostic tests”. Under these categories, we will further provide data on the article’s characteristics, including the total number of studies, types of study design, year of publications, and key findings. 

Secondly, we will use tables and figures to present the results in an organized manner, in line with the objectives and the review’s scope. We will also provide charts showing results as distribution of studies by year, area of intervention (epidemiological, clinical, interventional, or diagnostic), and research methods (if applicable).

Finally, the meaning of the results will be elaborated in the light of research and practice to help us identify gaps in the PD research in Romania. 

The results presentation will be further refined toward the end of the review, when we will have a greater awareness of the contents of the included studies. 

### 2.6. Quality Assessment

The present review’s scope is to map evidence that has been produced in the area of PD in Romania. We will not search for the best available evidence to answer a particular question related to the disease. The main distinction between scoping reviews and systematic reviews is that the scoping reviews provide an overview of the existing evidence, regardless of the quality of the included studies [8]. Therefore, we will not provide a formal assessment of the methodological quality of the included studies. 

## 3. Discussion

A scoping review is, in our case, the most appropriate approach, allowing for a broad research question. Therefore, a descriptive mapping of the literature body on PD in Romania is the best-suited review method.

The scoping review will enable us to draw conclusions based on the findings, including clear answers to our questions and objectives. We will also identify the gaps in research in this field in Romania, providing details on key implications for research and further need for primary research and/or systematic reviews in this area [6,8].

We will also detail key findings that can be used to inform practice. Nonetheless, providing implications for practice may be limited, as no methodological quality appraisal of the included studies will be made [8]. Additionally, as in scoping reviews, no rating of quality or level of evidence is produced, and practice recommendations will not be graded.

The results will be discussed in the context of current international literature, practice, and policy. We will also discuss any potential limitations of the sources included in the scoping review.

Finally, we will provide an overall conclusion based on the results, matched with our objectives and questions.

## 4. Conclusions

The publication of the present protocol offers our future scoping review a clear and transparent methodology, helping us avoid possible problems while performing the review.

To the best of our knowledge, there has been no previous systematic review on PD in Romania. We aim to present a complete picture of PD in Romania covering key aspects of the disease. Therefore, the outcomes of our study will be of interest to clinicians and other healthcare providers, policy-makers, and public health researchers. Furthermore, the present scoping review can be used as a precursor for further systematic reviews or meta-analyses with narrower questions assisting in developing and confirming inclusion criteria and research questions.

## Data Availability

Not applicable.
